# Orexin receptor antagonists attenuate acute stress-induced disruptions in sleep states

**DOI:** 10.3389/fnins.2026.1898419

**Published:** 2026-09-07

**Authors:** Kayla Lilly, Stephanie M. Perez, Daniel J. Lodge

**Affiliations:** 1 Department of Pharmacology and Center for Biomedical Neuroscience, UT Health San Antonio, San Antonio, TX, United States; 2 South Texas Veterans Health Care System, Audie L. Murphy Division, San Antonio, TX, United States

**Keywords:** footshock stress, orexin receptor antagonists, orexins (hypocretins), sleep disturbance, suvorexant

## Abstract

Post-traumatic stress disorder (PTSD) is characterized by symptoms including hypervigilance, intrusive memories, and sleep disturbance. Insomnia and nightmares are among the most common and clinically important sleep-related symptoms in PTSD, yet effective and well-tolerated pharmacotherapies remain limited. Suvorexant, a dual orexin receptor antagonist (DORA) approved for insomnia, represents a mechanistically distinct approach to traditional hypnotics. The orexin/hypocretin system is composed of two wake-promoting neuropeptides, orexin A and orexin B, which signal through orexin 1 (OX_1_R) and orexin 2 receptors (OX_2_R) to regulate arousal and sleep–wake transitions. The adaptive stress response, which is canonically disrupted in PTSD, is also moderated by the orexin system. Here, we used an acute inescapable footshock paradigm to disrupt sleep in adult male Sprague Dawley rats and evaluated whether orexin receptor antagonism restores sleep after stress. Systemic administration of a dual orexin receptor antagonist (suvorexant), an OX_1_R-preferring antagonist (SB334867), and an OX_2_R-selective antagonist (EMPA), partially restored REM sleep and attenuated increases in NREM sleep following footshock stress exposure. We also examined the relative contribution of OX_1_R and OX_2_R to these effects at the doses used. Although all antagonists increased time spent in REM after footshock stress, attenuation of the stress-induced increases in NREM and reductions in sleep-onset latency were most evident following dual receptor blockade. These findings demonstrate that orexin receptor antagonism can mitigate acute stress-induced alterations in sleep state allocation and sleep latency, and support further investigation of DORAs for stress-related sleep disturbance.

## Introduction

Post-traumatic stress disorder (PTSD) is a psychiatric disorder that can develop after exposure to a traumatic event and is characterized by symptoms that include intrusive memories, avoidance, negative alterations in mood and cognition, and hyperarousal (Department of Veterans Affairs). Sleep disturbance is also a prominent and clinically important feature of PTSD. Self-report studies estimate that up to 90% of individuals with PTSD experience insomnia-related symptoms and that a substantial proportion experience recurrent nightmares (Koffel et al., 2016; Brownlow et al., 2020; So et al., 2023). Sleep is critical for emotional regulation and for the processing of emotional experiences and memories (Koffel et al., 2016; Walker and van der Helm, 2009). As poor sleep can both reflect and exacerbate other dimensions of PTSD, insomnia, nightmares, and other nocturnal symptoms may contribute to a debilitating feedback cycle that worsens daytime function and treatment outcomes (Koffel et al., 2016; So et al., 2023; Germain, 2013; Zhang et al., 2019; Lappas et al., 2024). More broadly, exposure to severe stress can disrupt sleep and increase vulnerability to psychiatric illness, leading to increased research into the neurobiological mechanisms underlying stress-induced changes in sleep architecture (Pawlyk et al., 2008).

Treatment of PTSD-related sleep disturbance has commonly relied on pharmacotherapies, including sleep-promoting medications (Lappas et al., 2024; De Crescenzo et al., 2022; Lie et al., 2015). Benzodiazepines and non-benzodiazepine hypnotics such as zolpidem (“Z-drugs”) can improve insomnia symptoms, but concerns about residual sedation, tolerance, dependence, cognitive impairment, and misuse are particularly relevant in PTSD populations (Lappas et al., 2024; Lie et al., 2015; Samara, 2022; McCauley et al., 2012). Prazosin, an alpha-1 adrenergic receptor antagonist, is commonly used for PTSD-associated nightmares; however, its effects on global PTSD symptoms and overall sleep quality are mixed (Lappas et al., 2024; Lipinska et al., 2016; Raskind et al., 2018; Mellman et al., 2022). Consistent with these limitations, the U. S. Department of Veterans Affairs guidelines emphasize trauma-focused psychotherapies, specifically cognitive-behavioral therapy for insomnia, as first-line treatments and highlight the lack of research examining insomnia-focused treatments in PTSD (Brownlow et al., 2020; Department of Veteran Affairs, D. O. D, 2017). Suvorexant, an FDA-approved medication for insomnia characterized by difficulty with sleep onset and/or maintenance (Lie et al., 2015; Rhyne and Anderson, 2015; Herring et al., 2016), has emerged as a promising candidate for trauma-related insomnia (Mellman et al., 2022). In a clinical trial of suvorexant for trauma-related insomnia, both placebo- and medication-treated conditions improved, limiting detection of a medication-specific effect. Nonetheless, the findings supported continued evaluation of dual orexin receptor antagonists (DORAs) in this population (Mellman et al., 2022).

Suvorexant is one of three clinically approved DORAs, which are mechanistically distinct sleep-promoting agents (Lie et al., 2015; Mellman et al., 2022; Rhyne and Anderson, 2015; Coleman et al., 2017). The orexin system, also known as the hypocretin system, is a central regulator of arousal and sleep–wake stability (Inutsuka and Yamanaka, 2013b; Inutsuka and Yamanaka, 2013a). Orexin A and orexin B are produced by neurons in the lateral hypothalamic area and signal through two G-protein-coupled receptors, OX1R and OX2R (Inutsuka and Yamanaka, 2013b; Inutsuka and Yamanaka, 2013a; Elam et al., 2021). Genetic or experimental disruption of orexin signaling produces marked abnormalities in sleep–wake regulation in rodents, and loss of orexin signaling is strongly linked to human narcolepsy (Chemelli et al., 1999; Hara et al., 2001; Willie et al., 2003; Peyron et al., 2000; Nishino et al., 2000; Thannickal et al., 2000). These findings establish orexin signaling as a key regulator of wakefulness and a rational therapeutic target for insomnia. The orexin system is also highly conserved across mammals, supporting translational studies from rodents to humans (Coleman et al., 2017; Gotter et al., 2012; Winrow and Renger, 2014; Brisbare-Roch et al., 2007). Suvorexant was developed to promote sleep by blocking orexin-mediated arousal rather than broadly enhancing inhibitory neurotransmission, thereby lowering the threshold for transition from wakefulness to sleep (Coleman et al., 2017).

Orexin neurons are responsive to stress-provoking stimuli, and hyperactivity of the orexin system is associated with anxiety states (Johnson et al., 2012; Johnson et al., 2010). Further, evidence suggests orexin receptor antagonism promotes anxiolytic effects (Johnson et al., 2012; Panhelainen and Korpi, 2012). Given orexin signaling contributes to both vigilance and coordination of the adaptive stress response, it may represent an important mechanism linking acute stress exposure to subsequent changes in sleep (Johnson et al., 2012; Gotter et al., 2012). Inescapable footshock produces persistent behavioral and physiological consequences, including altered sleep, exaggerated fear, increased anxiety- and startle-related responses, and changes in glucocorticoid signaling (Elam et al., 2021; Van Dijken et al., 1992; Pawlyk et al., 2008; Pawlyk et al., 2005). Here, we used this acute stress paradigm to examine whether systemic administration of suvorexant, the OX1R-preferring antagonist SB334867, or the OX2R-selective antagonist EMPA attenuated stress-induced changes in sleep measured using wireless electroencephalography and electromyography in freely moving rats. Given the complementary role of both receptor subtypes in regulating arousal and vigilance (Gotter et al., 2012), we posited that dual blockade with suvorexant would be the most effective in the context of stress. We found that footshock stress altered baseline sleep stage time, including decreased REM and increased NREM, and modestly increased latency to sleep onset. Following footshock stress, systemic administration of orexin receptor antagonists partially increased REM sleep and attenuated stress-related increases in NREM sleep. Sleep-onset latency was also reduced, with effects most consistent with OX2R antagonism and most robust after dual receptor blockade. Together, these findings support orexin receptor antagonists, especially DORAs such as suvorexant, as candidate treatments for sleep disturbances associated with acute stress.

## Materials and methods

All experiments were conducted in accordance with the National Institutes of Health Guide for the Care and Use of Laboratory Animals and were approved by the Institutional Animal Care and Use Committees at UT Health San Antonio and the South Texas Veterans Health Care System.

Animals. Adult male Sprague Dawley rats (approximately 4–6 months of age; 350–450 g; Envigo, Indianapolis, IN, USA) were individually housed on a reverse 12-h light/dark cycle with food and water available ad libitum and without enrichment. All surgical procedures were performed under general anesthesia using isoflurane in a semi-sterile environment. Wireless EEG/EMG headmounts (Pinnacle Technology, Lawrence, KS, USA) were surgically implanted at least 1 week before the start of recordings, with electrodes positioned over the frontal and parietal cortices (approximately A/P + 4.0, M/L + 3.0 and A/P -4.0, M/L − 3.0 from bregma, respectively) and EMG wires placed into a subcutaneous pocket overlaying the neck muscle. Electrodes were isolated and secured using dental cement. Ketoprofen (5 mg/kg; 0.4 mL, subcutaneously) was administered immediately following completion of surgery for analgesia, and post-operative monitoring to assess overall health, including activity level, grooming behavior, and signs of complication or infection, was performed twice per day for a minimum of 48 h. Subsequent monitoring was performed daily by both research and animal husbandry staff to ensure overall health of animals. Intervention criteria were established as any overt sign of illness such as immobility, piloerection, porphyrin staining, and/or labored breathing. No animals were removed from this study due to meeting of humane endpoint criteria.

Inescapable footshock stress. After 1 week of pre-stress recordings, all animals underwent inescapable footshock stress. Rats were placed in a metal conditioning chamber (30.5 × 25.4 × 30.5 cm) with a grid shock floor (Coulbourn Instruments, Whitehall, PA, USA), and a series of footshocks (15 shocks; 1 s; 0.8 mA) were administered over a 90-min session with a mean inter-trial interval of 6 min and approximately 20% variability (±1 min). Vehicle post-stress recordings were conducted within 30 min following the completion of footshock exposure.

**Figure 1:**
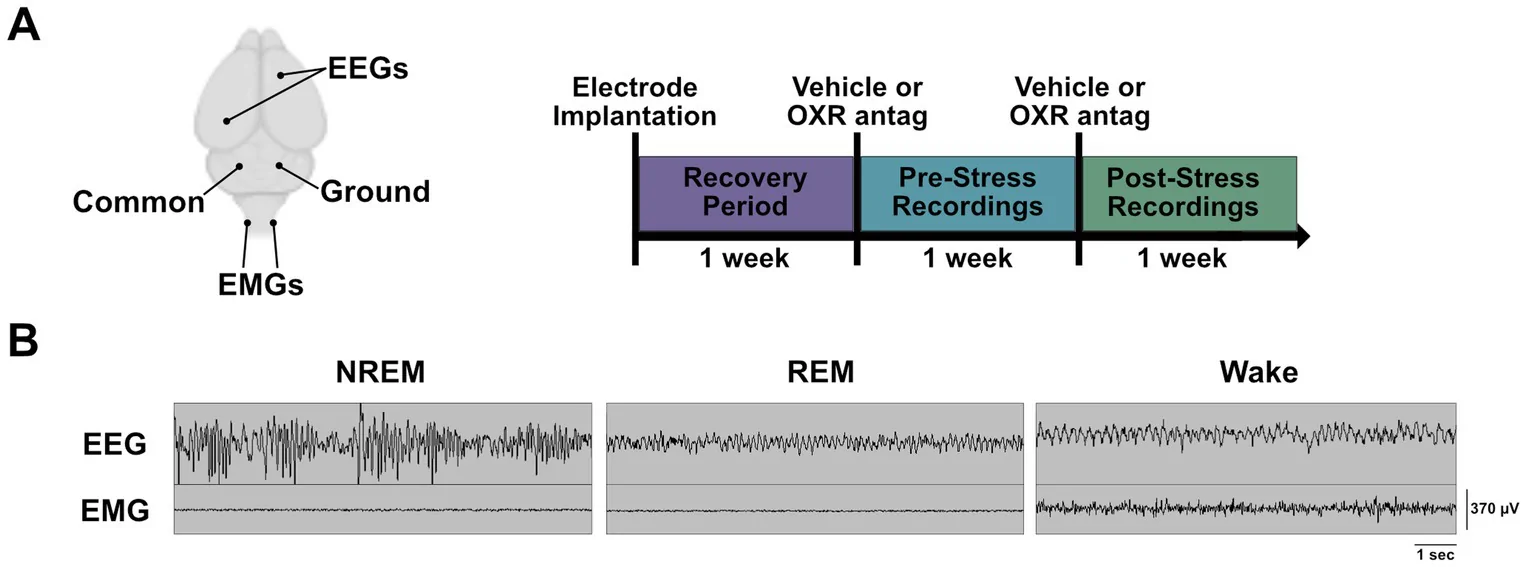
**(A)** Representative schematic of electrode placement and experimental timeline. **(B)** Representative EEG and EMG traces for NREM, REM, and wake states. NREM sleep was defined by slow-wave delta activity with little to no EMG activity, REM sleep by elevated theta relative to delta activity and low/paralyzed EMG activity, and wakefulness by little to no delta activity with prominent EMG activity. EEG, electroencephalogram; EMG, electromyogram; OXR, orexin receptor; footshock stress; NREM, non-rapid eye movement; REM, rapid eye movement.

Wireless EEG/EMG recordings. Wideband signals from implanted wireless EEG/EMG headmounts were acquired using Sirenia Acquisition software (Pinnacle Technology) for approximately 24 h per recording period. Sessions were started 1 h prior to the lights on cycle. Recordings were analyzed using Sirenia Sleep (Pinnacle Technology) with semi-automated cluster-based sleep scoring. Clusters were auto generated using standard frequency bands (delta: 0.5–4 Hz, theta: 5.5–8.5 Hz, alpha: 8–13 Hz, beta: 13–30 Hz, gamma: 35–44 Hz). Artifact exclusion was performed manually; the experimenter was not blinded to condition. Automated sorting limits manual error, though it is noted that complete accuracy is difficult to achieve with standardized scoring. However, total time spent in each of the sleep stages is comparable across conditions given the standardization of spectral frequencies. Wakefulness was defined by prominent EMG activity and little to no delta power, REM sleep by elevated theta relative to delta power and low/paralyzed EMG activity, and NREM sleep by slow-wave delta activity with low EMG activity. Latency to sleep onset was defined as the duration of time from the start of the recording to the first epoch of either REM or NREM sleep state. For drug-treatment experiments, orexin receptor antagonists (suvorexant, SB334867, or EMPA; 10 mg/kg; 0.4 mL, intraperitoneal) or vehicle (10% DMSO with 10% 2-hydroxypropyl-β-cyclodextrin in sterile water; 0.4 mL, intraperitoneal) were administered immediately before the start of recording. Drug doses were chosen based on previous experiments in our laboratory demonstrating effective reversal of aberrant mesolimbic dopamine system function as well as other pathophysiological phenotypes induced by footshock stress (Elam et al., 2021; Figure 1).

Experimental Timeline. Following surgical electrode implantation, all animals were given one full week to recover. The subsequent week, all animals were given vehicle for the baseline pre-stress recording, then given each of the three orexin receptor antagonists (suvorexant, SB334867, or EMPA) for the remaining pre-stress recordings. Injections were given 24 h apart and recordings initiated 1 h prior to the start of the lights-on period. During the third week, all animals underwent footshock stress. Vehicle was administered immediately following the completion of the stress session for the vehicle post-stress recording. Each day following, animals were given the antagonists in the same order they received them for the pre-stress recordings. Controls were within-subject, with all animals undergoing stress and receiving all treatments, and the order of drug administration was randomized and counterbalanced across animals. Thus, a total of 8 sessions were recorded per animal. A total of 10 animals entered the study. Individual recording sessions were excluded when less than 24 h of usable data were available because of power interruption or other technical error. Consequently, sample size varied across sleep-state and treatment comparisons. Exact sample sizes are reported in the corresponding figure legends.

**Figure 2:**
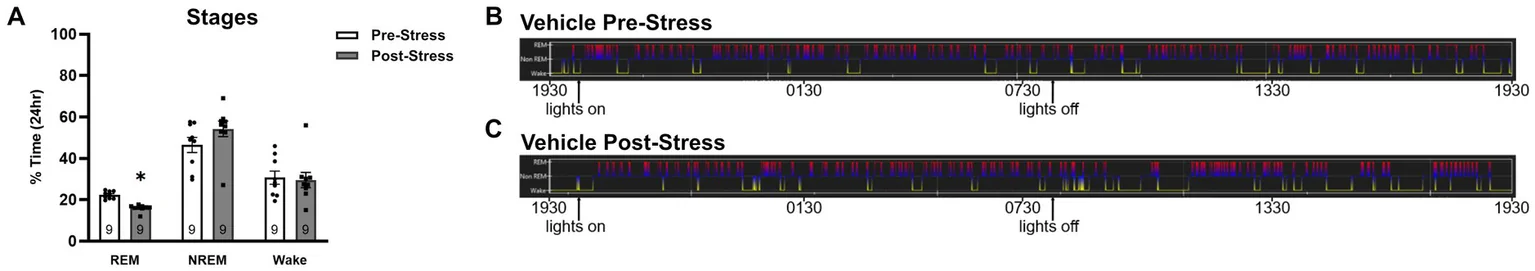
Inescapable footshock stress disrupts sleep state allocation under vehicle conditions. **(A)** Footshock stress altered normal sleep state times, decreasing REM sleep and compositionally increasing NREM sleep under vehicle conditions. Representative hypnograms comparing **(B)** vehicle pre-stress and **(C)** vehicle post-stress sleep patterns over 24 h illustrate altered sleep-state organization following inescapable footshock stress. NREM, non-rapid eye movement; REM, rapid eye movement. *Indicates significance between pre- and post-stress conditions within a given sleep stage.

**Figure 3:**
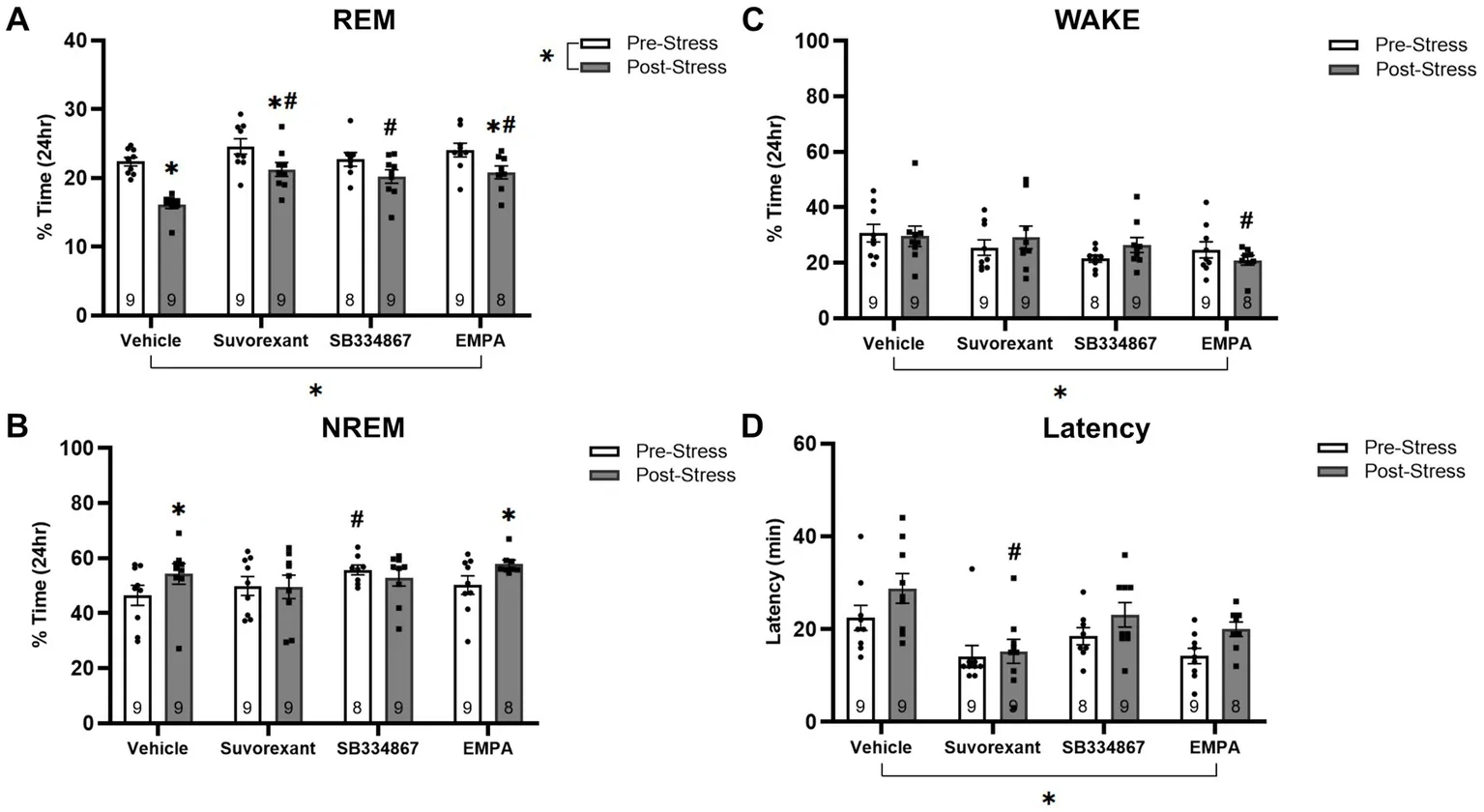
Systemic administration of orexin receptor antagonists partially restores stress-induced sleep state disruptions and reduces latency to sleep-onset. **(A)** REM sleep was reduced by stress under vehicle conditions and partially recovered after administration of suvorexant, SB334867, or EMPA. **(B)** NREM sleep increased following stress, but pre- and post-stress conditions did not differ after suvorexant or SB334867 treatment, suggesting attenuation of stress-induced increases in NREM sleep. **(C)** Time spent awake was not significantly affected by stress, but was by drug treatment, specifically by EMPA administration following stress. **(D)** Inescapable footshock stress moderately increased latency to sleep-onset. Suvorexant and EMPA modestly reduced sleep-onset latency, though only suvorexant significantly reduced sleep-onset latency, particularly under post-stress conditions. OXR, orexin receptor; NREM, non-rapid eye movement; REM, rapid eye movement. *Indicates significance between pre- and post-stress conditions within a given treatment. # Indicates significance between drug treatment and vehicle within either the pre- or post-stress condition. *Along *y*-axis indicates main effects of drug treatment. *Within legend indicates main effects of footshock stress.

Statistical analysis. Sleep stage data from Figure 2 were analyzed by paired t-tests (pre-stress x post-stress). For Figure 3, data were analyzed by two-way repeated measures analysis of variance (stress x drug treatment), followed by Holm-Sidak *post-hoc* tests when significant effects or interactions were observed. Data are presented as mean ± SEM, with n representing the number of animals per condition. One of the animals was removed from analysis due to technical failure during the pre-stress recording. Two sessions during which less than 24 h of recording were captured as a result of technical failure were also removed from the analysis in Figure 3 to prevent inaccurate comparisons. Stage data in Figure 2 are reflected as vehicle data in Figure 3 to separate the effects of stress from those of the antagonists. Graphs were generated using Prism (GraphPad Software, San Diego, CA, USA). Statistical analyses were performed using SigmaPlot (Systat Software, Richmond, CA, USA), and statistical significance was defined as *p* < 0.05. Of note, sleep states are compositional, and separate analyses do not account for the link between outcomes. Raw data will be made available upon reasonable request.

Materials. Suvorexant (Item No. 9002140; Cayman Chemical, Ann Arbor, MI, USA), SB334867 (Item No. 1960; Tocris, Minneapolis, MN, USA), and EMPA (Item No. 4558; Tocris) were prepared fresh daily and dissolved in 10% dimethyl sulfoxide (DMSO; Item No. D8418; Sigma-Aldrich, St. Louis, MO, USA) with 10% 2-hydroxypropyl-β-cyclodextrin (Item No. 16169; Cayman Chemical) in sterile water.

## Results

### Inescapable footshock stress disrupts baseline sleep states

To evaluate the impact of inescapable footshock stress on sleep states, animals were injected with vehicle and 24-h recordings of wideband EEG and EMG signals were acquired and analyzed using a three-state sleep classification approach. Consistent with previous work using shock-based stress models (Pawlyk et al., 2008), exposure to footshock stress altered baseline sleep patterns (Figure 2). Specifically, footshock stress decreased the percentage of time spent in REM sleep (Figure 2A; paired t-test; t = 9.541, *p* < 0.001). This was accompanied by a compositionally dependent, though not statistically significant, increase in NREM sleep (t = −2.217, *p* = 0.058). Total wakefulness was not significantly affected by stress (t = 0.318, *p* = 0.759), suggesting that footshock stress altered time spent in the sleep states rather than simply reducing overall sleep time. Representative hypnograms illustrate the redistribution of sleep states following stress (Figures 2B,C).

### Systemic administration of orexin receptor antagonists restores stress-induced disruptions in REM and NREM sleep

Given the role of orexin signaling in sleep–wake regulation, we examined whether orexin receptor antagonism could normalize sleep disturbances produced by stress. To evaluate the relative contributions of OX1R and OX2R, animals received the OX1R-preferring antagonist SB334867, the OX2R-selective antagonist EMPA, or the dual antagonist suvorexant. Main effects where stress decreased (two-way RM ANOVA; *F*_(1,69)_ = 29.799, *p* < 0.001) and drug treatment increased time spent in REM (two-way RM ANOVA; *F*_(3,69)_ = 14.856, *p* < 0.001) were observed for total REM sleep (Figure 3A). Under pre-stress conditions, treatment with suvorexant (Holm-Sidak *post-hoc* test; t = 2.694, *p* = 0.058), SB334867 (t = 0.158, *p* = 0.875), and EMPA (t = 2.054, *p* = 0.131) did not significantly alter time in REM. However, following stress, *post-hoc* analyses showed partial recovery following administration of all antagonists (suvorexant: t = 6.304, *p* < 0.001; SB334867: t = 5.068, *p* < 0.001; EMPA: t = 4.999, *p* < 0.001). These findings indicate partial normalization of REM sleep with orexin receptor antagonist administration. Consistent with the previous analysis, stress increased NREM sleep under vehicle = conditions (Figure 3B; Holm-Sidak *post-hoc* test; t = 2.361, *p* = 0.025). NREM percentage did not differ between pre- and post-stress conditions with suvorexant (t = 0.105, *p* = 0.917) or SB334867 (t = 0.831, *p* = 0.413), but did with EMPA (t = 2.136, *p* = 0.041). Of note, SB334867 increased NREM (Holm-Sidak *post-hoc* test; t = 2.773, *p* = 0.048) relative to vehicle treatment under pre-stress conditions. This suggests that dual antagonism is preferred to attenuate, though not fully normalize, the stress-induced NREM alteration. Percent wakefulness was not significantly affected by stress (Figure 3C; two-way RM ANOVA; *F*_(1,69)_ = 0.159, *p* = 0.701), however, main effects of drug treatment were observed (*F*_(3,69)_ = 5.013, *p* = 0.007), particularly following footshock stress. *Post-hoc* analysis revealed that EMPA (Holm-Sidak *post-hoc* test; t = 3.012, *p* = 0.025) decreased wakefulness, while SB334867 (t = 1.037, *p* = 0.665) and suvorexant (t = 0.146, *p* = 0.885) did not significantly alter wakefulness in the post-stress condition. Significant interactions between stress and treatment were observed on REM sleep (two-way RM ANOVA; *F*_(3,69)_ = 4.755, *p* = 0.011), but not NREM (two-way RM ANOVA; *F*_(3,69)_ = 2.269, *p* = 0.109) or wakefulness (two-way RM ANOVA; *F*_(3,69)_ = 1.779, *p* = 0.181).

### OX2R antagonism improves latency to sleep onset

Although footshock stress did not significantly alter total wakefulness, we next examined whether stress affected the latency to sleep onset. While not statistically significant, stress moderately increased sleep-onset latency (Figure 3D; two-way RM ANOVA; *F*_(1,69)_ = 4.762, *p* = 0.060). Because suvorexant promotes sleep onset clinically by attenuating orexin-mediated arousal (Coleman et al., 2017), we tested whether orexin receptor antagonism reduced latency to sleep onset following stress. A significant main effect of drug treatment was observed that overall reduced latency (Figure 3D; two-way RM ANOVA; *F*_(3,69)_ = 10.050, *p* < 0.001). SB334867 did not significantly alter sleep-onset latency under either pre- (Holm-Sidak *post-hoc* test; t = 0.983, *p* = 0.552) or post-stress (t = 1.825, *p* = 0.208) conditions. Contrastingly, suvorexant (t = 2.683, *p* = 0.059) and EMPA (t = 2.648, *p* = 0.054) partially reduced latency under pre-stress conditions. Following stress, suvorexant significantly reduced sleep-onset latency (Holm-Sidak *post-hoc* test; t = 4.365, *p* < 0.001). No interaction was observed between stress and treatment (two-way RM ANOVA; *F*_(3,69)_ = 0.574, *p* = 0.638). These results indicate that OX2R antagonism contributes strongly to sleep initiation, but that dual OX1R/OX2R antagonism may be more effective for reducing sleep-onset latency under stressed conditions.

## Discussion

Sleep disturbance is a prominent feature in PTSD and other stress-related disorders and can contribute to persistent hyperarousal, impaired emotional regulation, and poorer clinical outcomes (Koffel et al., 2016; Germain, 2013). Nightmares are trauma-related re-experiencing symptoms, while insomnia and difficulty returning to sleep may reflect persistent hyperarousal and hypervigilance (Spoormaker and Montgomery, 2008). Although sleep problems were historically considered secondary symptoms, they often persist, require targeted treatment, and can influence PTSD severity and recovery (Koffel et al., 2016; De Crescenzo et al., 2022; Lappas et al., 2024). Unfortunately, commonly used sleep-promoting agents, including benzodiazepines and non-benzodiazepine hypnotics, carry risks that are particularly relevant in individuals with PTSD (Lappas et al., 2024; Lie et al., 2015; Samara, 2022; McCauley et al., 2012). Orexin signaling provides a mechanistically distinct target because orexin neurons promote arousal and stabilize sleep–wake transitions (Inutsuka and Yamanaka, 2013b; Inutsuka and Yamanaka, 2013a). Further, orexins moderate the stress response (Johnson et al., 2012), suggesting that orexin receptor antagonism may be a beneficial approach to attenuating sleep disruption in the context of stress. Here, we used the footshock model to induce stress-related sleep abnormalities and examined whether orexin receptor antagonists normalize these disruptions. The present findings support the orexin system as a therapeutically relevant target for stress-associated sleep disturbance.

Sleep architecture can be differentially altered by stress and other physiological states (Han et al., 2012; Hall et al., 2000; Fuller et al., 1997; Edwards et al., 2010; Zapalac et al., 2024). In PTSD, objective sleep findings are heterogeneous across studies and may vary with trauma history, symptom profile, comorbid sleep disorders, and medication exposure (Koffel et al., 2016; Germain, 2013; Straus et al., 2015; van Wyk et al., 2016). Some studies report increased sleep fragmentation (Koffel et al., 2016; Harvey et al., 2003), while meta-analytic findings have described greater light sleep, reduced slow-wave sleep, and increased REM density in PTSD (Koffel et al., 2016; Kobayashi et al., 2007). In the present study, NREM sleep was analyzed as a single state rather than divided into human-like N1, N2, and N3 sub-classifications. Thus, the observed stress-induced compositionally dependent increase in NREM sleep cannot distinguish between increased lighter NREM sleep and changes in deeper slow-wave sleep. As contemporary perspectives emphasize the importance of consolidated, physiologically organized sleep for emotional memory processing and affective regulation (Walker and van der Helm, 2009), treatments that improve sleep onset, sleep maintenance, and sleep architecture may be preferable to treatments that broadly suppress specific sleep stages. Suvorexant, the first DORA approved by the FDA for insomnia, was developed for difficulty with sleep onset and/or sleep maintenance (Lie et al., 2015; Rhyne and Anderson, 2015; Herring et al., 2016). The present study aimed to induce stress-disrupted sleep changes to characterize the broad effects of targeting the orexin system and situate orexin receptor antagonism, particularly dual blockade, as an efficacious treatment approach during stress-sensitized states. To better determine how footshock stress may disrupt both the amount and organization of sleep, future analyses separating NREM substages, REM bout number, REM bout duration, and sleep fragmentation will be important for determining which components of sleep architecture are most sensitive to footshock stress and most responsive to orexin receptor antagonism. An additional consideration is that the pathophysiological phenotypes produced by the footshock stress paradigm are long-lasting (Nishimura et al., 2022; Rajbhandari et al., 2018; Pawlyk et al., 2008; Pawlyk et al., 2005), though the duration of the sleep alterations are not tested in this study. It is possible that sleep assessments across the 4 days post-footshock stress exposure may differ due to potential recovery from sleep disruption, thereby influencing the perceived effects of antagonist treatment. By counterbalancing drug treatment order, some of these effects can be mitigated, however, since vehicle treatment was always administered prior to the first post-stress recording, the possibility that the stress effect on sleep was attenuated or washed out by the second recording cannot be discounted. Thus, determining the extent to which footshock stress-disrupted sleep persists is optimal for further analysis.

Orexin neurons in the lateral hypothalamus project widely throughout the brain, consistent with the broad role of orexin signaling in arousal, autonomic function, reward, and sleep–wake stability (Inutsuka and Yamanaka, 2013b; Inutsuka and Yamanaka, 2013a; Marcus et al., 2001). Several orexin-innervated regions contribute to sleep-state regulation, including cholinergic and non-cholinergic nuclei in the brainstem and basal forebrain, as well as monoaminergic arousal systems such as the locus coeruleus (Inutsuka and Yamanaka, 2013b; Inutsuka and Yamanaka, 2013a; Marcus et al., 2001; Mieda et al., 2011). OX1R and OX2R show partially overlapping but distinct anatomical distributions, supporting complementary roles in sleep–wake regulation (Marcus et al., 2001, Mieda et al., 2011). Genetic studies indicate that disruption of OX2R produces more severe sleep fragmentation than disruption of OX1R (Inutsuka and Yamanaka, 2013b; Willie et al., 2003; Mieda et al., 2011; de Lecea and Huerta, 2014). However, findings from other studies show that the wakefulness-to-NREM transition depends upon OX_2_R, while dysregulation and suppression of REM emerge through contributions from both OX_1_R and OX_2_R (Inutsuka and Yamanaka, 2013b; Mieda et al., 2011). Together, these findings suggest that the two subtypes dually stabilize sleep/wake cyclicity, with greater input from OX_2_R as the dominant receptor in regulating sleep.

The present findings are consistent with this receptor-specific organization, where modest reductions in sleep-onset latency were observed with suvorexant and EMPA, but not SB334867, supporting a stronger role for OX2R in sleep initiation regardless of stress. Under stress conditions, however, only suvorexant significantly improved latency to sleep onset, suggesting that dual receptor blockade may provide broader efficacy in a stress-sensitized state. Partial restoration of REM sleep and attenuation of stress-induced NREM changes were observed across antagonist treatments, suggesting that blockade of either receptor subtype can influence stress-altered sleep state allocation. A caveat that dilutes the interpretation of receptor contribution in this study is the variability in pharmacokinetics between the orexin receptor antagonists used. As only one drug dose was tested, variance in receptor engagement as a result of underlying differences in potency and brain penetrance (Malherbe et al., 2009) cannot be ruled out as contributing to the differential effects observed. In particular, EMPA is known to require higher doses to produce adequate brain exposure (Malherbe et al., 2009). Thus, a weak EMPA effect, such as that seen with NREM, may be attributed to insufficient central receptor engagement at this dose rather than an inability of OX_2_R alone to produce a therapeutic effect. Though our data suggest dual orexin receptor blockade is most effective in the context of stress, the utilization of a dose–response to determine more comparable effects would strengthen this claim. In addition, EMPA is described as OX_2_R-selective (Malherbe et al., 2009), while off-target effects of SB334867 have been reported (Gotter et al., 2012) that indicate it is OX_1_R-preferring rather than truly selective, although selectivity profiles have consistently found roughly 45- to 50-fold selectivity for OX_1_R over OX_2_R (Gotter et al., 2012).

Interestingly, while REM sleep is largely decreased following stress, stress effects on NREM sleep are more heterogeneous within the literature, with type, duration, and timing of stress impacting outcomes (Pawlyk et al., 2008). Among shock stress paradigms, Pawlyk et al. details a variety of studies which mostly tested stress during early morning hours or within the first several hours of the lights on period (Pawlyk et al., 2008). While some found no changes or mild decreases, other studies found increases in NREM throughout either the light or dark phase (Pawlyk et al., 2008). Notably, general increases in NREM during the light phase were seen when stress exposure was closer to the start of light phase and increases during the dark phase when stress was implemented several hours after lights on (Pawlyk et al., 2008). Here, we detected compositionally dependent increases in NREM following footshock stress exposure within two 2 h before dark phase, though whether this was driven more by changes during the light or dark period needs to be examined further. Importantly, only EMPA significantly altered total wakefulness specifically after exposure to footshock stress, indicating that these treatments reorganized sleep state allocation and sleep initiation rather than simply increasing total sleep time. This distinction may be clinically relevant because an ideal treatment for stress-related sleep disturbance would improve the timing and quality of sleep without producing excessive residual sedation. However, given that locomotor activity was not directly measured in this study, the present findings cannot distinguish residual sedation or motor effects of orexin antagonism.

Finally, while it has been noted that prevalence of PTSD is elevated in women (Kessler et al., 1995; Grafe and Bhatnagar, 2020), it is believed that this sexual dimorphism is unrelated to non-associative fear sensitization which is largely modeled by the footshock stress paradigm that, importantly, does not exhibit sex differences (Nishimura et al., 2022). However, it is also evident that women are more likely to experience sleep disturbances (Bush et al., 2025) and that sleep in male and female mice is differentially affected by social defeat stress, a form of psychological stress (Bush et al., 2025). Further, orexin system expression is higher in females, and increased orexin activity may underly sex differences in the stress response (Grafe and Bhatnagar, 2020). Thus, a limitation of this study is the exclusion of females, leaving female-specific effects of OXR antagonist treatment relatively unknown. Repetition with the inclusion of sex as a variable would bolster the ability to generalize these findings to the broader population experiencing stress-associated sleep disturbances.

In summary, these data highlight the therapeutic potential of targeting orexin-mediated arousal to restore sleep–wake balance after acute stress. The limitations and risks associated with traditional sleep-promoting medications are particularly relevant in PTSD, prompting the search for safer and mechanistically distinct approaches. The orexin system regulates wakefulness and sleep-state transitions across mammals, making it a strong translational target. This study demonstrates that orexin receptor antagonism can improve sleep onset and partially restore disruption of sleep states in the context of acute stress, supporting further evaluation of DORAs for stress-associated sleep disturbances.

## Data Availability

The raw data supporting the conclusions of this article will be made available by the authors, without undue reservation.
